# Landscapes on Prevention Quality Indicators: A Spatial Analysis of Diabetes Preventable Hospitalizations in Portugal (2016–2017)

**DOI:** 10.3390/ijerph17228387

**Published:** 2020-11-12

**Authors:** Andre Ramalho, Mariana Lobo, Lia Duarte, Julio Souza, Paulo Santos, Alberto Freitas

**Affiliations:** 1 MEDCIDS-Department of Community Medicine, Information and Health Decision Sciences, Faculty of Medicine, University of Porto, 4200-319 Porto, Portugal; nanalobo@gmail.com (M.L.); psantosdr@med.up.pt (P.S.); 2 CINTESIS-Centre for Health Technology and Services Research, 4200-319 Porto, Portugal; juliobsouza1990@gmail.com; 3 Department of Geosciences, Environment and Land Planning, Faculty of Sciences, University of Porto, 4169-007 Porto, Portugal; liaduarte@fc.up.pt; 4 Earth Sciences Institute (ICT), Faculty of Sciences, University of Porto, 4169-007 Porto, Portugal

**Keywords:** diabetes mellitus, prevention quality indicators, avoidable admissions, geographic information system, public health

## Abstract

Preventable hospitalizations due to complications of diabetes mellitus (DM), represented by the related prevention quality indicators (PQI), are ambulatory care-sensitive conditions that can be prevented and controlled through effective primary health care (PHC) treatment. It is important to reduce mortality and promote the quality of life to diabetic patients in regions with higher hospitalization rates. The study aims to analyze the results of the DM age-sex-adjusted PQI, by groups of health centers (ACES), distributed in the Portuguese territory. The most representative PQI at a national level were identified, and the trends were mapped and analyzed. Also, it presents the ACES with the highest age-adjusted rates of avoidable hospitalizations for DM. The absolute number of preventable hospitalizations for all DM complications in Portugal has decreased by 20%, thus passing from the rate of 79 in 2016 to 65.2/100,000 inhabitants in 2017. Despite the improvement in results for PQI 03, 20 of 48 ACES that were above the national 2017 median rate in 2016, achieved better results the following year, and for the overall preventable diabetes hospitalizations (PQI 93) only 11 out 39, revealing the need for further studies and PHC actions to improve the diabetic quality of life.

## 1. Introduction

Diabetes Mellitus (DM) is certainly one of the non-communicable diseases that represent a major challenge in global health in recent decades, counting for some 1.6 million deaths annually [1,2]. In Portugal, according to the World Health Organization data (WHO), DM affected 13.3% of the population between 20–70 years old and was responsible for 5% of all deaths. The national health ministry promotes the monitoring and prevention of DM through of the National Diabetes Prevention and Control Program [3,4]. The promotion of integrated care for diabetic patients in the system has been evolving since 2006, with horizontal integration of the health and social assistance sectors, through the creation of the long-term care network of service providers. Integrated Diabetes Units were created to manage better the complexities of this condition, a service provided by specialists. The monitoring and achievement of better results in the quality indicators in primary health care have also been driven by incentives for the providers to have more comprehensive and effective coordination of care.

Despite all efforts to prevent complications from the disease, in recent years there has been an increasing trend in the prevalence of diabetes, mainly type 2 DM (t2DM) non-insulin dependent patients, counting for one of the main causes of mortality in European Union (EU). According to Institute for Health Metrics and Evaluation data, in 2017, Portugal presented the highest death rate from t2DM among Western European countries (31.91 deaths per 100,000 inhabitants) in 2017, followed by Cyprus (28.45 per 100,000 inhabitants), Italy (24.76 deaths per 100,000 inhabitants) and Malta (23.59 per 100,000 inhabitants).

Reducing mortality and promoting the quality of life of people living with diabetes is extremely important, especially in regions with higher rates of hospitalization. The prevention quality indicators (PQI) can be used with hospital discharge data to identify the quality of care for ambulatory care-sensitive conditions (ACSC) [5,6,7,8]. These population-based indicators were proposed by the Agency for Healthcare Research and Quality (AHRQ) [9] under the premise that good outpatient care can substantially prevent the need for hospitalizations, more serious complications of the disease and associated sequelae.

Even with the common difficulties in integration of levels of care, there is a strong need to join efforts to achieve the goals responsibly [10,11]. Primary health care (PHC) can lead to better health outcomes with lower costs and higher equity, as the cornerstone for providing health promotion and enhance accessibility to health care providers. A high-quality PHC can only be achieved through a systematic approach, seeking to understand the added value for the patient during their path in the system, whether it focus on the cure, disease prevention or health promotion.

Although they are based on hospitalization data, the PQIs can reveal information about the community health system or services outside the hospital environment, indirectly measuring the quality of primary health care (PHC) [12,13,14,15,16]. The integration of the spatial dimension in epidemiological investigations can offer an opportunity for conducting descriptive analyzes that bring interesting information to obtain new perceptions about how the data fit spatially to the prevention/control PHC actions and the causal processes under investigation [17,18,19]. In addition, demonstrating the results graphically can also show locations where public policies should compose more consistent actions for continuous improvement of their results. Although the number of available georeferenced databases has increased substantially and their cost has relatively decreased, the often substantial variation in quality between and within spatial databases remains a problem and, therefore, access to complete and updated metadata is particular importance when working with spatial data.

The Portuguese national health system has made efforts to improve its system, but disparities in the distribution of primary care facilities are still an important issue. Many regions present low coverage of the population by family doctors, representing real barriers to access and increased waiting times for assistance. Official data show that Portugal had 6848 general and family physicians in 2016–2017 biennium, and some difficulty to allocate health providers to the most remote areas [20].

In Portugal, since 2005 reform, PHC is based on health center groups (ACES), with administrative autonomy, composed by several units, including family practice, nursing and public health. ACES aim to guarantee the provision of primary care to the population in Portugal, covering the entire country, taking into account the number of living people, the settlement structure, the rate of ageing and the accessibility to the referral hospital, in dependence of five large regional administration (Norte, Centro, Lisboa e Vale do Tejo, Alentejo e Algarve).This study aim is to assess the geographical differences in the distribution of PQI in PHC, and the evolution of results in the 2016–2017 biennium. The identification of the PHC public services groupings with less favorable performances across regions adds the relevant result to the current awareness of diabetes primary care in Portugal, from which more in-depth actions and analyses may emerge and oriented by physicians, health researchers and public policymakers for diabetes.

## 2. Materials and Methods

### 2.1. Study Design and Variables

This study is a retrospective observational analysis of secondary data from the Portuguese national hospital databases for diabetes avoidable hospitalizations.

The period analyzed was the 2016–2017 biennium. It analyzes avoidable hospitalizations due to DM, represented by the PQI age-adjusted rate. These include the AHRQ DM-related PQIs (*n* = 5): PQI 01-Diabetes Short-Term Complications; PQI 03-Diabetes Long-Term Complications; PQI 14-Uncontrolled Diabetes; PQI 16 - Lower-Extremity Amputation Among Patients with Diabetes; PQI 93 - Prevention Quality Diabetes Composite (PQI 01, PQI 03, PQI 14, PQI 16).

### 2.2. Measurement

The prevention quality indicators have been computed according to AHRQ specifications, using the inpatient hospital data available through the national hospital morbidity database, which contains inpatient and outpatient hospitalizations data occurring in all public hospitals in mainland Portugal to determine the numerator. The Portuguese public hospitals account for 79% of all inpatient discharges and represent 72% of all inpatient hospital beds [21] The information regarding diagnoses and medical procedures is coded according to the International Classification of Diseases (ICD-9-CM and ICD-10-CM/PCS). The only inpatient discharges data covering the years 2016 and 2017 were used for this study, since these datasets are the latest available. The 2011 population census was used to determine the PQI denominators. Direct standardization was implemented to obtain age-sex-adjusted PQI using the 2011 Portugal census population as the standard population [20,21,22,23,24]. Directly standardized rates represent rates that would have existed if the population under study had the same age distribution as the “standard” population. Therefore, by being summary measures adjusted for differences in age-sex distributions, these rates afford more appropriate comparisons across the Portuguese ACES. ACES are grouping of essential PHC public services with administrative autonomy, consisting of several functional units, aiming to guarantee the provision of primary care to the population in Portugal, covering the entire country, taking into account the number of living people, the settlement structure, the rate of ageing and the accessibility to the referral hospital.

### 2.3. Data Analysis

#### 2.3.1. Relative Performance of the DM Prevention Quality Indicators across ACES

**Figure 1:**
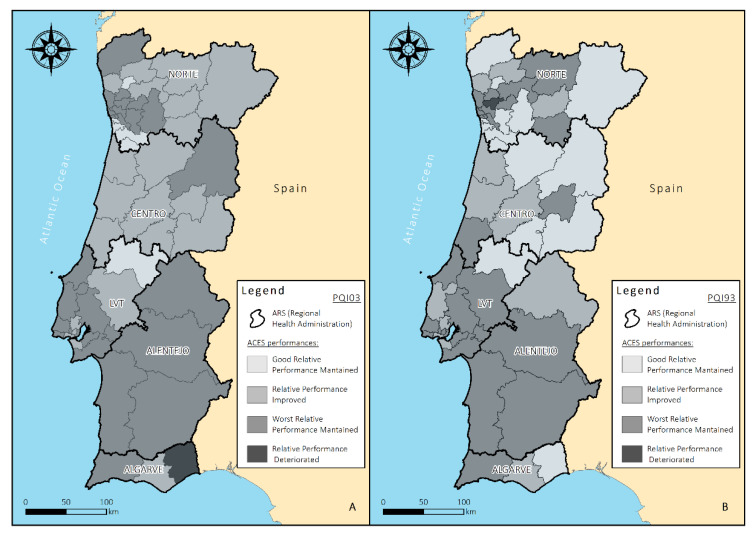
Geographic representation by ACES and Regional Health Administration areas—trend scenarios for the prevention quality indicators age-adjusted rate. (**A**) PQI 03-Diabetes Long Term Complications Admission Rate; (**B**) PQI 93-Diabetes Prevention Quality Indicator Composite.

Preventable hospitalization rates observed in Portuguese ACES were summarized using the median and interquartile range per year analyzed since the PQI values were not normally distributed across ACES. The ACES (*n* = 55) were distributed across regions, 24 of them in the Norte region, nine in the Centro region, 15 in the Lisboa e Vale do Tejo region, four in the Alentejo and three in the Algarve (Figure 1). The median PQI value observed in 2017 was used as a cutoff point, one per PQI, to classify ACES in terms of their performance in each year: better performing ACES (PQI > cutoff); worse performing ACES (PQI < cutoff).

To compare the ACES preventable hospitalizations rates from 2016 to 2017, four possible scenarios were inferred: ACES that maintained throughout the biennium a preventable hospitalization rate lower than the 2017 median rate; ACES that decreased the preventable hospitalization rate relative to median 2017 rate; ACES that maintained throughout the biennium a preventable hospitalization rate higher than the 2017 median rate; and ACES that increased the preventable hospitalization rate relative to median 2017 rate. The former two scenarios are favorable, whereas the latter two scenarios are unfavorable. The most representative PQI scenarios within preventable hospitalizations and the composite PQI (which considers all hospitalizations due to diabetes complications) were analyzed and represented geographically in more detail.

#### 2.3.2. Geographic Distribution of Relative Performance across ACES in Portugal

For a better perception and discussion of the results, in 2017 the Norte, Centro and Lisboa and Vale do Tejo regions were the most populous regions in the country exhibiting the highest number of hospital beds per capita (more than 310 beds/100,000 inhabitants). In contrast, Alentejo and Algarve had considerably lower population sizes and beds per capita (217 and 256 beds/100,000 inhabitants, respectively). However, Alentejo had a population density that is 3 times smaller than the next smallest population density region (Centro) and 11 times smaller than the highest population density region (Lisboa e Vale do Tejo). Unemployment rates ranged from 5.9% in Centro to 9.3% in Norte, and retention and dropout rates in primary education are smaller in Norte (9.3%), Centro (4.8%) and LVT (6.5%) when compared to Alentejo (7.1%) and Algarve (7.3%) (Table 1) [21,22].

In order to assess the distribution of these preventable hospitalization rates for DM, an analysis was performed to understand the complications of diabetes PQI with greater representativeness in the composite and the results of the composite indicator itself (PQI 93) between ACES and the health administrative areas. A spatial representation of the trend scenarios for the 2016–2017 biennium of the PQI 03 and PQI93 results was made through the ArcGIS 10.5.1 software. This Geographic Information System (GIS) software is maintained by the global company “Environmental Systems Research Institute” (Esri) and allows to perform spatial analysis (Esri, Sacramento, CA, USA). It can be used as a support decision system allowing the visualization of information resulted from a large volume of data, in a multi-scale perspective [23,24,25,26,27]. It thus assists in the identification and direction of the problem solving and improvement of public policies, including public health [17,18,23].

The shapefile is representing the geographical influence area of each ACES and obtained from the National Health System (in WGS 84 (World Geodetic System 1984, EPSG:4326). The projected coordinate system used in the map was the ETRS89, PT TM06 European Terrestrial Reference System 1989 Portugal Transverse Mercator 2006, EPSG: 3763), currently the global reference system recommended by EUREF (European Reference Frame). Subsequently, the shapefile related information was combined with data from PQI 03 and PQI 93. The polygons on the map represent the ACES, which are classified according to the 2017 results scenario compared to the 2016 results, with the median of the PQI values’ age-adjusted rate as the evaluation cut-off. The map was divided in 5 health administrative regions: Norte, Centro, Alentejo, Lisboa e Vale do Tejo and Algarve.

## 3. Results

The results of the age-sex-adjusted rates for diabetes prevention quality indicators described below were summarized in Table 2, and the data for the scenarios are presented in full in the tables of Appendix A.

### 3.1. Relative Performance Results of the DM PQI across ACES

In 2016, PQI 01 - Short-term Diabetes Complications Admission Rate, was the second most representative indicator in the group of indicators, accounting for 22.56% of preventable DM hospitalizations (*n* = 1928) in Portugal and corresponding to a median of 17.11 hospitalizations/100,000 pop. across ACES. In 2017, the results were lower, with 471 fewer hospitalizations. The national median age-sex-adjusted admission rate for PQI 01 in 2017 was 13.7 hospitalizations/100,000 pop. The result for 2016 varied between 6.04 and 52.32 hospitalizations/100,000 pop. in 2016 and between 3.41 and 30.33 hospitalizations/100,000 pop. the following year. In 2016, 4 ACES were outliers for presenting results above the upper limit (quartile 3 + 1.5 × IQR), ACES Alto Tâmega e Barroso (52.32 hospitalizations/100,000 pop.), ACES Amadora (43.95 hospitalizations/100,000 pop.), ACES Baixo Alentejo (36.98 hospitalizations/100,000 pop.) and ACES São Mamede (35.98 hospitalizations/100,000 pop.) (Appendix A). There were no outliers for this PQI in 2017. In the best scenarios, there were 11 ACES below the national median in both years (<13.70 hospitalizations/100,000 pop.), two of which (ACES Pinhal Interior Norte and Santo Tirso/Trofa) had an upward trend. Seventeen ACES in 2016 were above the 2017 national median but showed improvement in 2017 staying below the median.

PQI 03 - Long-term Diabetes Complications Admission Rate, was the indicator with the highest impact within the group over the two years, accounting for 4012 preventable diabetes hospitalizations in 2016 and 2639 in 2017 (34.22% reduction). The national median age-sex-adjusted admission rates were 40.26 and 26.25 hospitalizations/100,000 pop., in 2016 and 2107 respectively. In 2016, the result for this rate varied between 16.84 and 99.04 hospitalizations/100,000 pop. in 2016, and between 6.79 and 55 hospitalizations/100,000 pop. for the following year. In 2016, 1 ACES was outlier for presenting results above the upper limit (quartile 3 + 1.5 × IQR): ACES Baixo Alentejo (198.52 hospitalizations/100,000 pop.). In 2017 there were no outliers for this PQI. For the scenario analysis, we used the median of 2017 (26.25/100,000 pop.) as an assessment cut-off for the two years. In the best scenarios, six ACES were below the national median in both years for this PQI (<26.25 hospitalizations/100,000 pop.), also with two ACES (ACES Pinhal Interior Norte and Santo Tirso/Trofa) with in an upward trend. Twenty one ACES had improved their rates from 2016 to 2017, i.e., the rates decreased to values below the median in the second year.

The PQI 14 - Uncontrolled Diabetes Admission Rate, in 2016, registered the lowest median admission rate in the group of indicators (10.5 hospitalizations/100,000 pop.). However, with 320 hospitalizations more than the previous year, in 2017, the median admission rate was 12.78 hospitalizations/100,000 pop., making this PQI the second most representative value in the group of indicators in the second year. The ACES rates varied between 0 and 40.11 hospitalizations/100,000 pop. in 2016 and between 1.7 and 47.97 hospitalizations/100,000 pop. in the following year. The only ACES with a value equal to zero hospitalizations in the first year was ACES Pinhal Interior Sul; the others had values above 0.89 hospitalizations/100,000 pop. In 2016, two ACES were outliers for presenting results above the upper limit (quartile 3 + 1.5 × IQR), ACES Pinhal Litoral (38.76 hospitalizations/100,000 pop.) and ACES Cova da Beira (40.11 hospitalizations/100,000 pop.). In 2017, two were the outliers: ACES Estuário do Tejo (47.97 hospitalizations/100,000 pop.) and, once more, ACES Cova da Beira (39.33 hospitalizations/100,000 pop.). When we evaluated the PQI considering the median of 2017 (12.78 hospitalizations/100,000 pop.), in the most favorable scenarios, we found 14 of the 22 ACES with results below the median of 2017 in the two years presents a worsening of the values of the rates from one year to the other. In 2016, only six ACES that were above the 2017 median representing an improvement in their results in the second year. In the unfavorable scenario, 13 out of 15 ACES which had a PQI higher than the median in both years also worsened their results. The two of the 15 ACES that were in this scenario but had a slight improvement were ACES Cova da Beira and ACES Pinhal Litoral. There were 12 ACES that raise more concern because had results below the 2017 median in the first year and were above in the second. Particularly, PQI rate in ACES Arco Ribeirinho increased from 10.5 to 32 hospitalizations/100,000 pop. in 2016 and 2017, respectively, the highest rate increase for this PQI among ACES.

With much lower rates and less representativeness within the group of indicators assessed, the median PQI 16 - Lower-Extremity Amputation among Patients with Diabetes Admission Rate, in 2016, was 12.10 hospitalizations/100,000 pop. In 2017, it accounted for 202 admissions less than the previous year corresponding to a national median admission rate of 9.6 hospitalizations/100,000 pop. Despite this improvement, it was observed that in 2017 this PQI represented 16.51% of all DM preventable hospitalizations, while in 2016 it represented only 15.54%. In 2016, we observed only 1 ACES with results above the upper limit (quartile 3 + 1.5 × IQR), the ACES Baixo Alentejo (40.59 hospitalizations/100,000 pop.). In the following year, the only ACES above the upper limit was the ACES Estuário do Tejo (24.63 hospitalizations/100,000 pop.). As for the favorable trend scenarios for this indicator, 13 ACES remained below the national median of 2017 for both years evaluated. However, five of these 13 ACES showed an upward trend, despite still having results below the assessment value, with particular attention to ACES Médio Tejo, which reached 9.59 hospitalizations/100,000 pop. in 2017. The largest improvement in this scenario was ACES Pinhal Interior Norte, which rate more than halved from one year to the next (from 7.2 to 2.8 hospitalizations/100,000 pop.). Still, as a favorable scenario, 15 ACES that had rates above the 2017 national median, reduced their rates from 2016 to 2017. Of these 15 ACES the most significant improvement was observed in ACES Gerês/Cabreira, which went from 19.1 to 6.46 hospitalizations/100,000 pop. The unfavorable scenarios show 24 ACES with rates above the median in the two years, 13 of them with worse values in 2017 compared to 2016. The ACES posing the most critical concern in this group is the ACES Estuário do Tejo, which increased from 16.4 to 24.23 hospitalizations/100,000 pop. Moreover, the ACES that, despite being above the national median rate in the two years, improved its results was ACES Baixo Alentejo, decreasing from 40.59 to 20.47 hospitalizations/100,000 pop. Three ACES had good results in 2016 (below the 2017 national PQI rate median) and had higher rates in 2017 (ACES Algarve Sotavento—7.90 to 13.80/100,000 pop., ACES Cascais—7.66 to 14.96/100,000 pop., and ACES Alto Minho—8.22 to 12.41/100,000 pop.).

PQI 93, which refers to the age-sex-adjusted rate of admissions composite for diabetes complications, that is, contains in its numerator the sum of admissions due to all four situations of preventable hospitalization for the year 2016 (8545 hospitalizations) and 2017 (6819 hospitalizations). In 2017, therefore, there was a reduction of 1726 preventable DM hospitalizations (20.2% less than in the first year of analysis). It shows that there was a positive result in reducing the overall diabetes hospitalizations in Portugal. For this PQI, ACES Estuário do Tejo was once again the only ACES with values above the upper limit (quartile 3 + 1.5 × IQR), of 198.56/100,000 pop. in 2016. In 2017 there were no outliers. PQI-93, in general, presented more favorable than unfavorable results. Fifteen ACES presented PQI93 below the 2017 national median rate of preventable hospitalizations for DM in the two years. Of these 15, only three had worse results in 2017 compared to the previous year despite maintaining rates below the median. These were: ACES Algarve Sotavento (from 38.76 to 41.82 hospitalizations/100,000 pop.), ACES Alto Minho (from 57.19 to 57.22 hospitalizations/100,000 pop.) and ACES Gaia (from 54.33 to 56.78 hospitalizations/100,000 pop.). Twelve ACES had results above the 2017 median for the rate in 2016 but improved their results in the next year, with rates below 65.20 hospitalizations/100,000 pop., with a more significant improvement in the ACES Gerês/Cabreira (from 89.59 to 34.41 hospitalizations/100,000 pop.) and ACES São Mamede (from 100.8 to 52.6 hospitalizations/100,000 pop.). In unfavorable scenarios, 27 ACES were above the cut-off established for evaluation. Of these, 23 showed a slight improvement. In contrast, the other four ACES were not only above but also had worse results from one year to another: ACES Alentejo Litoral (from 75.42 to 83.64 hospitalizations/100,000 pop.), ACES Arrábida (from 87.36 to 91.60 hospitalizations/100,000 pop.), ACES Vale do Sousa Norte (from 68.21 to 72.06 hospitalizations/100,000 pop.) and ACES Estuario do Tejo, with the worst result rising from 106.01 to 120.58 hospitalizations/100,000 pop. The only ACES that had results below the 2017 national median in 2016 and that in the next year performed above, was ACES Santo Tirso/Trofa, from 56.43 to 67.73 hospitalizations/100,000.

For the biennium, the PQI rates interquartile ranges (IQR) are reduced, and the min-max values are closer from one year to other. The performance quadrants allow us to identify the ACES that are farthest from the defined cutoff, and their performance in the two years relative to the median 2017.

### 3.2. Geographic Distribution of Relative Performance across ACES in Portugal

PQI 03 deals with long-term complications of type 1 and type 2 diabetes-related to complications with renal, ophthalmic, neurological, peripheral, and other unspecified manifestations. As shown before, this PQI had the highest representativeness in the composite indicator (PQI 93). Figure 1 shows the spatial representation of the ACES referring to the trend scenario of the age-adjusted rate, for the PQI 03 and PQI 93.

It is essential to find out what the problem is and where it will be assessed. This study demonstrates that analyzing the composite in isolation can influence the interpretation of results. The map requires additional information so that it can target actions at the right time and to the right population. Figure 1A shows the results of the relative performance of ACES for the prevention quality indicator 03 (diabetes long term complications admission rate) for the biennium and 1-B for the prevention quality indicator 93 (diabetes complication composite rate).

As for PQI 03, the overall spatial representation showed a greater proportion of ACES (22 of 55, 49% of ACES) in the scenario characterized by a worst relative performance maintained throughout the two years, represented by 12 of the 24 ACES in the Norte Region, one of the nine ACES in the Centro Region, nine of the 15 ACES in the Lisboa e Vale do Tejo and one of the three ACES in the Algarve. The second most representative scenario within this PQI was that characterized by a relative performance improvement, with 21 of the 55 ACES (38.2%), eight of which belong to the Centro region, seven to the Norte region, five to Lisboa and Vale do Tejo and one to the Algarve. Only six ACES (five from the Norte region and one from the Lisboa e Vale do Tejo region) presented a good relative performance-maintained scenario. A single ACES presented a relative performance deteriorated (below the 2017 median in 2016 and above in 2017)—ACES Sotavento in the Algarve Region. The ACES with the highest preventable hospitalization rate in this PQI in 2017 was the ACES Arrábida, located in the region of Lisboa e Vale do Tejo (55 hospitalizations/100,000 inhabitants). ACES with a more substantial improvement from one year to the next (despite remaining above the median) was ACES Sintra also located in the Lisboa e Vale do Tejo region (from 56.95 to 26.25 hospitalizations/100,000 inhabitants) (Appendix A).

The results for PQI 93, a composite that include all preventable hospitalizations due to diabetes in the numerator (PQI 01, PQI 03, PQI 14 and PQI 16), show a higher proportion of ACES in the scenario characterized by a worst performance maintained throughout the two years (Figure 1B). Twenty six of the 55 national ACES are found in this scenario (47.3% of the total), encompassing 11 of the 24 ACES in the Norte Region, two of the nine ACES in the Centro Region, 10 of the 15 ACES in the Lisboa e Vale do Tejo, three of the four ACES in Alentejo and one of the three ACES in the Algarve. The second most representative scenario within this PQI was that of good relative performance-maintained scenario, with 15 ACES present (nine in the Norte region, four in the Centro region, one in Lisboa e Vale do Tejo and one in the Algarve). Twelve ACES were on the scenario of relative performance improved (three Norte, three Centro, four Lisboa and Vale do Tejo, one in Alentejo and one in Algarve). Only one ACES’s relative performance deteriorated with respect to preventable hospitalizations due to all complications of diabetes (ACES Santo Tirso/Trofa, in the North) which went from 56.43 to 67.73 hospitalizations/100,000 inhabitants). The ACES with the highest preventable hospitalization rate in this PQI in 2017 was the ACES Estuário do Tejo, located in the region of Lisboa e Vale do Tejo (120.6 hospitalizations/100,000 inhabitants). ACES with a more substantial improvement from one year to the next (despite remaining above the 2017 median) was ACES Baixo Alentejo, located in the Alentejo region (from 198.52 to 89.95 hospitalizations/100,000 inhabitants).

For the analyzed period, ACES Sotavento in the Algarve region, was the only ACES that worsened within its scenario, as it already had results above the national rate median and deteriorated further, that is, from 20.78 in 2016 to 28.43/100,000 inhabitants in 2017. Interestingly, the map shows us that, for this same region, the results made up in PQI 93 (Figure 1B) are better performing. The same dynamic did not happen in the northern region, where ACES Santo Tirso/Trofa, presented to PQI 03 maintained a poor performance (above the median in the two years), and still worsened within its scenario (from 30.18 to 45.89/100,000 inhabitants). For PQI 93, there is a more significant contribution of PQI 03 in the burden of preventable DM hospitalizations. The darkest colour represents this ACES in Figure 1B.

In the Algarve region, we see a similar pattern of classification for two ACES, Barlavento and Central Algarve. The first maintained a poor performance for both indicators while ACES Sotavento, a relative performance deteriorated in PQI 03. However, for PQI 93 ACES Sotavento is in the category of good maintained performance. This result occurs since ACES Sotavento in PQI 01, and 14 had a good maintained performance with reduced rates from one year to the next. In contrast, PQI 03 and 16 had their performance deteriorated, being below the median in 2016 but above in 2017 (PQI03 = 28.43 and PQI16 = 13.82/100,000 inhabitants). Of the total of 439,617 inhabitants, 21.87% are elderly.

The Alentejo region is the region with the highest proportion of older adults patients who generally have multiple chronic conditions and are therefore more susceptible to long-term complications. It is, therefore, more targeted and personalized approaches to the chronic complications of DM that generate preventable hospitalizations (PQI03). In this region, however, there was an ACES that had its composite rate improved, ACES São Mamede, which went from 100.80 to 48.20/100,000 inhabitants (reduction of 47.8%). This region had this result in the composite as it managed to improve its performance in all indicators of complications (except PQI 03, where it remained slightly above the median in the second year, 26.35/100,000 inhabitants.

As for the Centro region, eight of the nine ACES had improved results for PQI 03, and ACES Guarda was the only one that maintained a poor result for the two years. ACES Guarda, despite maintaining a result above the median in the two years, showed improvement in its result from one year to the next (from 32.21 to 28.66). This region is the third most populous in the country and has the third-highest proportion of elderly in the regions (25.41). In the analysis of PQI 93, we see a lighter map, which represents that four out of 10 ACES had good results maintained, emphasizing that ACES Guarda could still improve its results especially in PQI 03. An ACES in PQI 93 in the region maintained poor results ACES Cova da Beira maintained a poor result, impacted not only by PQI 03 but also by PQI 14 and 16. Despite having responded to an improvement in their values from one year to the next, they maintained a poor performance in the biennium compared to national results.

The Lisboa e Vale do Tejo Region, the most populous region of the country, presented in PQI 03 a single ACES with good maintained performance, and a result also demonstrated in PQI 93 (ACES Medio Tejo). Five ACES had their results improved in PQI 03, with reduction of rates in all of them, in particular, ACES Lezíria, which went from 40.26 to 16.11/100,000 inhabitants). Although nine ACES maintained worse maintained results, all of them had reduced rates from one year to the other except for ACES Arrábida, the highest national rate 55/100,000 inhabitants.

In the North region, specifically further south of the region, we observed 12 ACES that maintained the results of their rates from one year to the other concerning PQI 03. However, of those seven ACES maintained rates below the median in the two years, but signalled a slight increase rate from one year to the next (ACES Alto Minho, Ave/Famalicão, Aveiro Norte, Baixo Tâmega, Feira e Arouca, Gaia, and Vale do Sousa Sul). ACES Santo Tirso/Trofa is the darkest area, previously described.Therefore, despite presenting better results in the year of 2016 among the groups of indicators assessed, the individual scenarios for ACES have individual trends that requires more considerable attention from services to diabetic patients (Appendix A).

## 4. Discussion

To the best of our knowledge, this is the first study that presents the distribution of preventable hospitalization rates for diabetes complications in Portuguese territory. It is also the first one to analyze these results through performance scenarios of trends.

Portugal has endeavored, through its national diabetes prevention and control program, to reduce not only rates of hospitalization due to complications but also mortality due to DM. Therefore, Portuguese PHC has been complying with the commitments signed with the World Health Organization and reaffirmed in the Declaration of St. Vicent [28] of which Portugal participated. Expertise in Primary care management of diabetes is naturally uneven but to identify those practices that need help to improve care necessitates methods to identify these sites.

The PQI values for DM established by AHRQ give us a direction for strengthening primary health care the main responsible for the management of treatment and follow-up of these patients. The absolute number of preventable hospitalizations for all diabetes complications in Portugal has decreased by 1726 hospitalizations (20%), thus passing from the rate of 79 to 65 hospitalizations/100,000 inhabitants. The PQI 03, which deals with long-term complications, was the most representative of the total number of preventable DM admissions, corroborating the natural history of the disease and reflecting control efforts at the primary level [29,30,31,32]. These long-term complications (despite being the leading causes of hospitalizations in the two years) had a significant reduction in the absolute number of preventable hospitalizations—from 4012 hospitalizations to 2639 (reduction of 34%). Despite the improvement in results for PQI 03, 20 of 48 ACES that were above the median of 2017 in 2016, achieved better results the following year, and for the composite of preventable diabetes hospitalizations (PQI 93) only 11 out 39, revealing the need for further studies and actions to improve the diabetic quality of life.

The best available evidence shows us that PQIs has been validated to assess the quality of health care in some countries [29,33,34,35,36,37,38,39,40,41]. However, these studies in their majority had comparisons more at the national level [33,34,35,36,37,38] than at the regional level [42,43,44] that are also diverse between countries. Some articles sought to identify predictors of access to PHC [43,44,45,46] which makes it difficult to directly compare the evidence already published with the study we have shown.

We recognize that the present study may have some limitations. First, it is important to remember that our study does not intend to assess barriers on access to health care services and predisposing factors of preventable hospitalizations for diabetes mellitus, but only to map an overview of the performance of primary health care regarding hospitalizations for complications of an ambulatory care sensitive condition in the biennium in question, in order to identify the groups of health centers that demand greater attention from health managers and professionals, with intensification of health promotion, disease monitoring actions, and future micro-analyzes at the level of the units belonging to these groupings.

Secondly, we were unable to consider many potential confounding factors. The high heterogeneity found in the rates of hospitalization for complications from DM may suggest differences in critical areas in the quality of primary health care. The indicators presented may be associated with some factors that are external to the health system, such as socioeconomic level, environmental conditions. lack of patient compliance with treatment, data not available for the studied aggregation of ACES [33,34,35,36,37,38,39,40,41]. ACES were used as a unit of analysis because in Portugal, the district health network is administered independently in accordance with national policy. Comparisons with other countries should be treated with great caution, as in previous studies the selected geographical units are varied [12,35,36,41]. Barriers to access care for diabetic patients, such as the geographical barrier or the absence or low availability of caregivers or family members to conduct to the consultations, especially older patients, even the existence of family health units can also be factors that can interfere with the results presented. Again, these data were also not available for analysis. Although the results presented are engaging in the sense of signaling the greatest need for intensification of disease prevention and control actions, we must exercise caution in interpreting the data as an absolute truth since the analysis period reported only to two years. Therefore, the methodology of this study must be applied later in a more extended period, in a prospective study.

This article brings added value in terms of knowledge about trends in the results of primary health care actions in the territory, concerning the unfavorable outcome (avoidable hospitalizations). It provides a spatial demonstration of the results, contributing to identify locations where public policies should compose more consistent actions of continuous quality improvement, like in access, follow up, continuity, treatment, health education, among others. Besides the analysis can be replicated, it can also be improved with more details at the local level, observing factors that can contribute to the improvement of results, such as associations with quality indicators of primary health care, socioeconomic and human resources allocated for treatment and monitoring of these patients. Furthermore, it may be the starting point for the analysis of other PQI rates such as the Chronic Obstructive Pulmonary Disease, Hypertension Admission Rate, among others. Several studies have been conducted and published in the literature regarding PQI, in many approaches, from the simplest to the most complex [32,47,48,49,50,51,52], that are possible allied methodologies for a more in depth comparative analysis, which can be reinforced and based on this method of identifying areas through their respective quadrants of performance scenarios, still to be performed by our group in Portugal. The analysis of quality indicators related to diabetes in PHC and their association with the variability of the results of preventable hospitalizations, in addition to the efficiency analysis of the resources applied in PHC, are ways to deepen this analysis, either individually in each ACES as per regions.

## 5. Conclusions

Our study identified a significant rate reduction from one year to the next, and PQI 03 (long-term diabetes complications) as the most representative from the total preventable hospitalizations in the two years. Despite the performance improvement in, we notice that many ACES at the national level remain with rates above the 2017 national median, both for PQI 03 and for the composite of preventable diabetes hospitalizations, revealing the need for further studies and actions to improve the diabetic quality of life. The benefit of the quadrant scenarios through spatial analysis method to identify low-performance primary care facilities has proved to be practical and a suitable visual method for signaling ACES with performance standards variation for diabetes complications indicators and their impact on the results of the composite PQI across ACES.

## Figures and Tables

**Table 1:** Regional Backgrounds (2016–2017).

Region	Area (km^2^)	Resident Population	Elderly Proportion	Physicians (per 100,000 pop.)	Hospital Beds (per 100,000 pop.)	Unemployment Rates (%)	Retention and Dropout Rates (Primary Education) (%)
Norte	21,278	3,5762,05	19.83	500	312.3	9.3	4.3
Centro	28,462	2,231,346	25.41	640	314.16	5.9	4.8
Lisboa e Vale do Tejo	11,633	2,833,679	22.95	470	380.9	8.2	6.5
Alentejo	31,551	711,950	27.8	390	216.9	8.4	7.1
Algarve	4997	439,617	21.87	290	256	7.3	7.6

**Table 2:** Prevention Quality Indicators Rates and Number of Hospital Admissions (2016–2017).

DM Prevention Quality Indicators	2016					2017				
	Admissions (*n*, %)		Age Adjusted Admission Rate			Admissions *(n,* %)		Age Adjusted Admission Rate		
		Median	Min	Max	IQR		Median	Min	Max	IQR
PQI 01-Diabetes Short Term Complications Admission Rate	1928 (22.56%)	17.11	6.04	52.32	8.4	1457 (21.37%)	13.7	3.41	30.33	9.26
PQI 03-Diabetes Long Term Complications Admission Rate	4012 (46.95%)	40.26	16.84	99.04	18.60	2639 (38.70%)	26.25	6.79	55.01	15.2
PQI 14-Uncontrolled Diabetes Admission Rate	1227 (14.94%)	10.50	0	40.11	11.84	1597 (23.42%)	12.78	1.70	47.97	11.66
PQI 16-Lower-Extremity Amputation among Patients with Diabetes Admission Rate	1328 (15.54%)	12.10	1.49	40.59	7.526	1126 (16.51%)	9.6	2.84	24.627	7.219
PQI 93-Diabetes Prevention Quality Composite Rate	8545 (100%)	79	38.76	198.52	36.78	6819 (100%)	65.2	32.32	120.58	30.76

## Appendix A

**Table A1:** Good relative performance maintained.

Good Relative Performance Maintained (Below the Median for both Years)														
PQI 01 (13.70/100,000 POP.)			PQI03 (26.25/100,000 POP.)			PQI 14 (12.78/100,000 POP.)			PQI 16 (9.60/100,000 POP.)			PQI 93 (65.20/100,000 POP.)		
ACES (*n* = 11)	2016	2017	ACES (*n* = 6)	2016	2017	ACES (*n* = 22)	2016	2017	ACES *(n* = 13)	2016	2017	ACES (*n* = 15)	2016	2017
SOTAVENTO	7.491	7.093	MÉDIO TEJO	20.696	21.723	ALENTEJO CENTRAL	6.580	9.077	BEIRA INTERIOR SUL	5.805	4.948	SOTAVENTO	38.762	41.823
BAIXO MONDEGO	10.794	5.481	AVEIRO NORTE	16.839	23.290	BARLAVENTO	1.230	12.394	PINHAL INTERIOR NORTE	7.196	2.835	BEIRA INTERIOR SUL	63.521	35.482
BAIXO VOUGA	12.690	11.889	BRAGA	22.900	9.259	ALGARVE CENTRAL	6.308	8.636	PINHAL INTERIOR SUL	1.488	2.977	DÃO LAFÕES	59.112	52.968
BEIRA INTERIOR SUL	13.637	5.668	ESPINHO GAIA	18.042	13.755	SOTAVENTO	3.966	3.347	LISBOA CENTRAL	7.420	6.604	GUARDA	63.702	63.108
DÃO LAFÕES	12.824	12.094	FEIRA E AROUCA	22.980	17.347	BEIRA INTERIOR SUL	10.758	5.828	MÉDIO TEJO	8.863	9.597	PINHAL INTERIOR SUL	46.632	40.500
GUARDA	11.078	3.405	GAIA	18.803	17.401	PINHAL INTERIOR SUL	0.000	11.659	BAIXO TÂMEGA	6.206	4.374	MÉDIO TEJO	61.483	61.311
PINHAL INTERIOR NORTE	6.040	7.762				ALMADA/SEIXAL	4.666	10.979	BRAGA	8.836	9.193	ALTO MINHO	57.194	57.223
PINHAL INTERIOR SUL	11.923	7.709				ARRÁBIDA	5.290	10.887	DOURO SUL	9.498	5.632	AVEIRO NORTE	45.101	44.040
ALTO MINHO	11.305	10.253				CASCAIS	7.881	10.848	ESPINHO GAIA	4.826	5.871	BAIXO TÂMEGA	52.477	45.862
AVEIRO NORTE	11.686	9.064				LOURES/ODIVELAS	11.769	11.567	FEIRA E AROUCA	9.174	8.443	BRAGA	52.846	33.841
SANTO TIRSO/TROFA	12.148	12.706				ALTO MINHO	4.461	8.894	MARÃO E DOURO NORTE	6.235	5.961	ESPINHO GAIA	58.547	48.888
						AVE/FAMALICÃO	5.391	6.955	NORDESTE	8.267	9.231	FEIRA E AROUCA	57.413	44.621
						AVEIRO NORTE	4.355	6.255	VALE DO SOUSA SUL	8.908	3.146	GAIA	54.333	56.778
						BAIXO TÂMEGA	3.227	4.692				NORDESTE	59.802	46.943
						BRAGA	6.432	1.696				VALE DO SOUSA SUL	60.988	59.245
						ESPINHO GAIA	10.268	6.450						
						FEIRA E AROUCA	5.765	7.744						
						GAIA	8.565	9.900						
						GERÊS/CABREIRA	12.332	5.646						
						NORDESTE	9.291	9.290						
						SANTO TIRSO/TROFA	7.427	4.358						
						VALE DO SOUSA SUL	3.636	9.027						

**Table A2:** Relative performance improved.

PQI 01 (13.70/100,000 pop.)			PQI03 (26.25/100,000 pop.)			PQI 14 (12.78/100,000 pop.)			PQI 16 (9.60/100,000 pop.)			PQI 93 (65.20/100,000 pop.)		
ACES (*n* = 17)	2016	2017	ACES (*n* = 21)	2016	2017	ACES (*n* = 6)	2016	2017	ACES (*n* = 15)	2016	2017	ACES (*n* = 12)	2016	2017
São Mamede	35.98	11.14	Algarve Central	29.92	14.63	Baixo Alentejo	21.36	6.01	São Mamede	12.34	7.46	São Mamede	100.80	48.20
Cova da Beira	19.57	13.26	Baixo Mondego	29.96	15.60	São Mamede	22.23	5.53	Barlavento	19.04	7.50	Algarve Central	66.57	47.23
Pinhal Litoral	15.68	12.60	Baixo Vouga	29.56	15.59	Baixo Mondego	27.31	12.78	Baixo Mondego	13.08	8.40	Baixo Mondego	84.09	39.37
Arco Ribeirinho	23.80	10.57	Beira Interior Sul	35.19	18.21	Pinhal Interior Norte	29.57	8.75	Baixo Vouga	16.00	7.18	Baixo Vouga	66.18	50.06
Lisboa Central	15.32	11.11	Cova da Beira	45.85	25.98	Lisboa Ocidental e Oeiras	13.11	11.65	Dão Lafões	10.11	6.12	Pinhal Interior Norte	81.23	32.32
Loures/Odivelas	22.04	9.20	Dão Lafões	28.15	20.96	Porto Ocidental	14.19	11.82	Pinhal Litoral	10.36	7.34	Almada/Seixal	79.04	60.79
Médio Tejo	17.14	13.70	Pinhal Interior Norte	33.03	13.26				Lisboa Norte	10.11	9.17	Lisboa Ocidental e Oeiras	90.57	63.86
Oeste Sul	18.33	7.61	Pinhal Interior Sul	33.22	19.64				Loures/Odivelas	14.65	6.54	Loures/Odivelas	82.03	45.62
Baixo Tâmega	15.02	7.31	Pinhal Litoral	49.42	18.42				Oeste Sul	19.74	9;36	Oeste Sul	86.03	61.11
Barcelos/Esposende	15.80	7.89	Almada/Seixal	40.74	24.23				Alto Tâmega e Barroso	10.51	7.01	Barcelos/Esposende	71.14	43.47
Braga	17.11	12.96	Lezíria	40.26	16.11				Aveiro Norte	11.31	6.40	Gerês/Cabreira	89.59	34.41
Douro Sul	17.80	10.10	Lisboa Norte	36.05	23.97				Gerês/Cabreira	19.10	6.46	Marão e Douro Norte	74.23	57.49
Feira e Arouca	16.02	11.47	Lisboa Ocidental e Oeiras	46.88	23.01				Porto Oriental	17.64	8.14			
Maia/Valongo	14.47	13.64	Loures/Odivelas	34.68	15.80				Vila do Conde/Póvoa de Varzim	13.12	9.41			
Marão e Douro Norte	16.27	6.19	Alto Ave	30.91	15.72				Vale do Sousa Norte	10.71	6.08			
Vila do Conde/Póvoa de Varzim	16.56	10.23	Alto Tâmega e Barroso	47.81	26.21									
Vale do Sousa Sul	21.78	10.72	Barcelos/Esposende	32.21	12.12									
			Douro Sul	29.53	25.71									
			Gerês/Cabreira	42.42	6.79									
			Marão e Douro Norte	34.95	25.70									
			Nordeste	29.69	14.63									

**Table A3:** Worse relative performance maintained.

PQI 01 (13.70/100,000 pop.)			PQI03 (26.25/100,000 pop.)			PQI 14 (12.78/100,000 pop.)			PQI 16 (9.60/100,000 pop.)			PQI 93 (65.20/100,000 pop.)		
ACES (*n* = 24)	2016	2017	ACES (*n* = 27)	2016	2017	ACES (*n* = 15)	2016	2017	ACES (*n* = 24)	2016	2017	ACES (*n* = 27)	2016	2017
Alentejo Central	21.637	19.511	Alentejo Central	64.125	48.226	Cova da Beira	40.108	39.332	Alentejo Central	11.914	10.805	Alentejo Central	99.523	83.644
Alentejo Litoral	14.480	21.303	Alentejo Litoral	49.683	36.872	Guarda	18.673	27.514	Alentejo Litoral	12.729	16.290	Alentejo Litoral	75.421	87.350
Baixo Alentejo	36.985	21.201	Baixo Alentejo	99.038	48.211	Pinhal Litoral	38.758	33.432	Baixo Alentejo	40.590	20.474	Baixo Alentejo	198.516	89.950
Barlavento	21.336	21.224	São Mamede	30.627	26.352	Estuário do Tejo	19.781	47.970	Algarve Central	19.736	10.612	Barlavento	76.886	69.389
Almada/Seixal	18.301	15.631	Barlavento	45.973	31.106	Lezíria	20.551	32.094	Cova da Beira	17.193	14.835	Cova da Beira	123.057	100.265
Amadora	43.948	27.003	Guarda	32.216	28.660	Lisboa Central	20.829	24.192	Guarda	10.225	11.785	Pinhal Litoral	109.994	72.845
Arrábida	21.659	18.535	Amadora	51.438	28.114	Lisboa Norte	16.738	23.525	Almada/Seixal	17.650	13.033	Amadora	116.684	91.031
Cascais	20.989	15.988	Arco Ribeirinho	57.131	30.982	Médio Tejo	16.298	19.122	Amadora	17.313	22.379	Arco Ribeirinho	101.125	90.664
Estuário do Tejo	25.732	23.944	Arrábida	50.843	55.016	Oeste Norte	19.060	26.796	Arco Ribeirinho	12.900	19.606	Arrábida	87.362	91.600
Lezíria	30.177	30.331	Cascais	42.218	38.245	Barcelos/Esposende	12.796	14.450	Arrábida	18.919	22.091	Cascais	76.314	75.856
Lisboa Norte	15.315	11.105	Estuário do Tejo	48.520	33.310	Douro Sul	23.868	26.491	Estuário do Tejo	16.434	24.627	Estuário do Tejo	106.010	120.580
Lisboa Ocidental e Oeiras	22.801	17.137	Lisboa Central	47.430	31.021	Maia/Valongo	20.975	30.019	Lezíria	16.114	13.600	Lezíria	107.362	93.348
Oeste Norte	26.245	22.133	Oeste Norte	42.968	30.844	Marão e Douro Norte	17.600	21.373	Lisboa Ocidental e Oeiras	12.095	10.842	Lisboa Central	96.097	76.816
Sintra	31.920	19.481	Oeste Sul	48.985	32.596	Porto Oriental	17.064	19.588	Oeste Norte	16.279	14.716	Lisboa Norte	85.637	74.814
Alto Ave	25.699	20.851	Sintra	56.954	26.254	Vila do Conde/Póvoa de Varzim	16.211	20.165	Sintra	22.981	20.888	Oeste Norte	98.262	92.667
Alto Tâmega e Barroso	52.316	21.092	Alto Minho	35.033	26.892				Alto Ave	11.814	13.100	Sintra	121.262	77.992
Ave/Famalicão	17.672	19.367	Ave/Famalicão	41.397	28.744				Ave/Famalicão	12.626	13.823	Alto Ave	73.751	68.676
Espinho Gaia	25.646	23.047	Baixo Tâmega	29.379	28.439				Barcelos/Esposende	16.960	11.751	Alto Tâmega e Barroso	118.881	76.155
Gaia	16.708	20.530	Gondomar	63.251	44.091				Gaia	9.740	12.447	Ave/Famalicão	72.139	68.662
Gerês/Cabreira	24.363	14.674	Maia/Valongo	58.348	52.825				Gondomar	13.871	16.325	Douro Sul	78.253	67.364
Gondomar	21.836	20.816	Matosinhos	43.408	38.068				Maia/Valongo	14.582	13.705	Gondomar	107.893	89.216
Matosinhos	16.680	14.022	Porto Ocidental	54.842	35.152				Matosinhos	16.442	16.457	Maia/Valongo	105.103	102,845
Nordeste	16.276	14.813	Porto Oriental	51.735	35.092				Porto Ocidental	13.185	15.797	Matosinhos	79.595	77.376
Vale do Sousa Norte	15.659	18.499	Vila do Conde/Póvoa de Varzim	47.680	30.950				Santo Tirso/Trofa	11.918	13.585	Porto Ocidental	93.292	75.038
			Santo Tirso/Trofa	30.178	45.894							Porto Oriental	90.306	80.079
			Vale do Sousa Norte	43.607	32.264							Vila do Conde/Póvoa de Varzim	90.383	67.097
			Vale do Sousa Sul	27.234	37.002							Vale do Sousa Norte	68.208	72.057

**Table A4:** Relative performance deteriorated.

PQI 01 (13.70/100,000 pop.)			PQI03 (26.25/100,000 pop.)			PQI 14 (12.78/100,000 pop.)			PQI 16 (9.60/100,000 pop.)			PQI 93 (65.20/100,000 pop.)		
ACES (*n* = 03)	2016	2017	ACES (*n* = 1)	2016	2017	ACES (*n* = 12)	2016	2017	ACES (*n* = 3)	2016	2017	ACES (*n* = 1)	2016	2017
Algarve Central	12.268	14.685	Sotavento	20.784	28.435	Alentejo Litoral	3.845	15.473	Sotavento	7.927	13.819	Santo Tirso/Trofa	56.432	67.729
Porto Ocidental	10.056	16.076				Baixo Vouga	8.851	13.592	Cascais	7.664	14.957			
Porto Oriental	10.054	14.008				Dão Lafões	10.738	16.903	Alto Minho	8.221	12.408			
						Amadora	7.362	13.927						
						Arco Ribeirinho	10.488	31.979						
						Oeste Sul	5.510	14.337						
						Sintra	12.527	13.258						
						Alto Ave	6.181	20.405						
						Alto Tâmega e Barroso	11.165	22.989						
						Gondomar	10.279	13.685						
						Matosinhos	5.968	13.222						
						Vale do Sousa Norte	0.891	15.523						
